# Einthoven Dissertation Prizes 2020

**DOI:** 10.1007/s12471-021-01601-7

**Published:** 2021-08-13

**Authors:** J. J. Piek

**Affiliations:** Department of Cardiology, Heart Centre, Amsterdam UMC, location AMC, University of Amsterdam, Amsterdam, The Netherlands

The dissertation prize is named after Willem Einthoven, a pioneer in cardiovascular medicine who recorded the first human ECG in 1902, for which he was awarded the Nobel prize in 1924. The annual Einthoven dissertation prize is an initiative of the Netherlands Heart Institute (NHI) and the Netherlands Society of Cardiology (NVVC) to select the top three cardiovascular theses published in the year 2020. The jury received a total of 30 PhD dissertations for selection. The ranking of the theses was based upon a combination of parameters that included the curriculum vitae of the candidate, the scientific originality of the PhD thesis and its relevance for the cardiovascular field. Moreover, several objective bibliometric parameters were used that included the number of articles in citation index journals, both in PubMed and the Web of Science (WOS), the number of citations in WOS, the Hirsch index, and finally the contribution of the candidate as first author. Based upon this evaluation the jury selected the following nominees: Vincent Aengevaeren (Radboudumc), Martijn Smulders (MUMC+) and Najim Lahrouchi (VUMC). The members of the jury were prof. P.A.F.M. Doevendans and prof. J.W. Jukema (Netherlands Heart Institute), prof. A.C. van Rossum (NVVC), dr. M.J.W. Götte (CVOI), dr. F.M.A.C. Martens (WCN) and dr. G. Veen (President *Concilium Cardiologicum*). The three candidates presented their PhD theses at the 5th DCVA Translational Cardiovascular Research Meeting in Utrecht, the Netherlands, on Thursday June 24, 2020. We congratulate the laureates for their excellent scientific work and their presentations during the meeting.

## Potential detrimental effects of exercise on the heart

The beneficial effects of exercise are indisputable. Nevertheless, there is discussion whether one can exercise too much. The aim of this thesis was to investigate the potential detrimental effects of (mainly endurance) exercise on the heart to answer the question: “*can exercise hurt the heart?”*. For this purpose, we investigated the presence and relevance of exercise-induced increases in cardiac biomarkers, long-term cardiac remodelling, myocardial fibrosis and coronary atherosclerosis in athletes.

We observed significant increases in cardiac biomarkers (BNP, troponin and sST2) following prolonged walking exercise and marathon running, but the underlying mechanisms of release and clinical relevance remained unclear. Therefore, we assessed the relationship between post-marathon cardiac troponin I concentrations and cardiomyocyte integrity using multiparametric cardiac magnetic resonance imaging. Intriguingly, higher post-marathon troponin concentrations correlated with a lower cardiomyocyte integrity (higher mean diffusivity of myocardial tissue water), suggesting that exercise-induced troponin elevations may result from ‘leaky’ cardiomyocytes. We also discovered that cardiac troponin I elevations above the 99th percentile independently predicted higher mortality and cardiovascular events in our longitudinal cohort study of the Four Days Marches. Thus, exercise-induced troponin increases may not be a benign physiological response to exercise, but an early marker of cardiovascular risk.

It is unclear how exercise can affect cardiac remodelling in certain cardiac diseases and if prolonged, intensive exercise alone can induce detrimental cardiac adaptation. First, we evaluated cardiac remodelling in Olympic athletes participating in 3 consecutive Olympic Games. We observed continued right heart remodelling after 4 years of intensive training, but no further changes occurred after 8 years. Also, no detrimental adaptations were found in these healthy young athletes. Second, we evaluated whether physical activity affects hypertrophic cardiomyopathy (HCM) genotype expression and disease characteristics. We found no association between physical activity volumes and genotype to phenotype transition in HCM gene carriers or a difference in disease characteristics across physical activity volumes among HCM patients. Nevertheless, the most active HCM patients were younger at the time of diagnosis and had a higher arrhythmic burden.

Furthermore, we evaluated the prevalence and pattern of myocardial fibrosis in athletes in a systematic review. We observed that myocardial fibrosis was more common in athletes compared with control cohorts, however, it was predominantly present in the interventricular septum and where the right ventricle joins the septum. These specific locations are likely less detrimental.

There are concerns that exercise might accelerate coronary atherosclerosis. Therefore, we investigated the relationship between physical activity and coronary atherosclerosis. We observed that the most active athletes had significantly higher coronary artery calcification (CAC) scores and prevalence of CAC and plaque compared with the least active athletes. However, the most active group had a lower prevalence of mixed plaques and more often had only calcified plaques compared with the least active group. Sport-specific analyses revealed that cyclists had a lower prevalence of atherosclerotic plaques and trended toward a lower prevalence of CAC compared with runners. Cyclists more often had only calcified plaques. Increased CAC amongst athletes may not necessarily reflect an increased risk for cardiovascular events similar to the general population since exercise promotes beneficial coronary adaptations and increased calcification may be associated with plaque stabilisation, which likely explains the significant reduction in cardiovascular events despite the presence of increased CAC in endurance athletes.

Findings from this thesis suggest that exercise *can* hurt the heart of certain vulnerable individuals, but the benefits of exercise far outweigh the harms for most individuals.

Vincent Aengevaeren, Department of Physiology, Radboudumc, Nijmegen, the Netherlands, (✉) Vincent.Aengevaeren@radboudumc.nl

## Diagnostic evaluation of chest pain—the role of non-invasive cardiac imaging

Chest pain is a symptom of a wide variety of diseases, ranging from a trivial ailment to more serious potentially life-threatening disease. Identifying the underlying aetiology can be challenging and additional diagnostic testing is often necessary. While considered crucial in patients with chest pain, ineffective use and overspending of testing must be avoided as much as possible. This thesis describes the role of non-invasive diagnostic testing in patients with acute and chronic chest pain.

### Acute chest pain

Acute chest pain is the most common presenting complaint in the emergency department. The troponin test result is often decisive for further management in patients with an inconclusive electrocardiogram. A normal high-sensitivity cardiac troponin T (hs-cTnT) value essentially rules out acute myocardial infarction (MI) with high confidence. In our observational study in 918 patients with acute chest pain and normal hs-cTnT levels, we observed an excellent 1‑year prognosis. Despite this very low risk of events, particularly in patients with an atypical history (86%), additional non-invasive (imaging) testing was performed in up to 50% of patients. Abnormal test findings and therapeutic interventions were infrequent in these patients, suggesting that the use of additional testing was not beneficial.

Hs-cTn assays have lower specificity to diagnose MI. Since its introduction, non-obstructive coronary artery disease (CAD) is more frequently observed at invasive coronary angiography in suspected MI. Ideally, unnecessary invasive procedures should be avoided as much as possible to prevent potential procedure-related complications, longer hospital length of stay and health care costs. We have performed a randomised controlled trial (CARMENTA) to investigate the clinical effectiveness of implementing cardiac magnetic resonance imaging (CMR) or computed tomography angiography (CTA) first compared with routine care in patients with suspected non-ST-elevation MI (NSTEMI). Non-obstructive CAD was observed in one third of patients. A novel strategy of implementing CMR or CTA first in the diagnostic process in NSTEMI reduced the number of invasive coronary angiograms and improved appropriate referral. In addition, CMR frequently identified an alternative diagnosis (e.g. myocarditis) in patients without CAD and both imaging-guided strategies showed a trend towards less 1‑year adverse events. The CARMENTA trial uniquely showed the gatekeeping potential of early CMR and CTA in this population.

In a two-centre trial in patients with proven MI, we studied different aspects of infarct healing with cardiac MRI. In contrast to prior knowledge, we found that T2-weighted hyperintensity may persist up to 6 months following MI, which questions whether T2 exclusively reflects oedema. Microvascular obstruction and increased myocardial wall thickness were more specific markers for acute MI. This study provided insight into the different underlying pathophysiological mechanisms of MI and proved that a multicomponent MRI approach determines infarct age more accurately than T2 alone.

### Chronic chest pain

Additional diagnostic testing is often performed to rule out CAD. The prognostic value of a test is particularly important if the test result is negative, as this should reassure the patient and physician. We have performed a meta-analysis of 165 studies (122,721 patients) that compared the prognostic value of a negative test result between all non-invasive cardiac tests in patients with suspected CAD. This study concluded that a negative test result yielded an excellent prognosis and was comparable between modalities after adjusting for differences in patient population.

Martijn Smulders, Department of Cardiology, Maastricht UMC+, Maastricht, the Netherlands, (✉) martijn.smulders@mumc.nl

## Dissecting genetic risk in common and rare inherited disorders

Susceptibility to the majority of human diseases is to a varying extent determined by genetic factors. Despite recent progress in our understanding of the genetic underpinnings of cardiovascular diseases, the genetic architecture and the underlying genetic factors for many of these disorders remain unknown, hindering clinical implementation and utility of genetic testing. This thesis addressed such knowledge gaps, focussing on both rare Mendelian disorders and common multifactorial traits. This led to new insights into their genetic architecture and uncovered novel genetic factors, some of which were also functionally investigated in cellular and animal models. This abstract presents some highlights of the thesis.

Work conducted in the first part provided insight into the genetic architecture of the long QT syndrome (LQTS). A genome-wide association study, which was conducted in 1781 patients with LQTS recruited internationally, provided unequivocal evidence for the role of common genetic variants in disease susceptibility. Of high clinical relevance, this work also demonstrated that ‘mutation-negative’ LQTS patients (i.e. those who do not harbour a rare pathogenic variant in the established LQTS genes), have a higher burden of common QT-prolonging alleles compared with mutation-positive patients, pointing to a likely complex inheritance in these patients.

The second part of the thesis sought to determine the role of post-mortem genetic testing in victims of sudden unexplained death. With the use of next-generation sequencing in 302 sudden unexplained death cases, we showed that the underlying genetic cause can be established in a clinically relevant proportion of cases (13%) and demonstrated that catecholaminergic polymorphic ventricular tachycardia and LQTS are responsible for most cases. Also, combining post-mortem genetic testing with the clinical evaluation of relatives was found to increase diagnostic yield. These findings are of clinical importance as the identification of the causal genetic factor in a victim crucially enables the identification of those relatives at risk, thus allowing for the timely implementation of preventive measures.

The third part of the thesis underscored the power of whole-exome sequencing in gene discovery for rare diseases. We identified bi-allelic loss-of-function variants in *PLD1* as the cause of severe congenital right-sided cardiac valve defects and neonatal cardiomyopathy. Functional studies on PLD1 pointed to abnormal endothelial mesenchymal transition, an established pivotal early step in valvulogenesis, as the likely underlying mechanism. In another study, we identified recessive variants in *POPDC2* as a cause of a novel arrhythmia syndrome presenting with hypertrophic cardiomyopathy and sinus node and atrioventricular node dysfunction.

In the fourth part, we conducted a large-scale study in up to 1.3 million individuals that combined exome array data in cohorts with, amongst others, UK Biobank data, to investigate the role of rare single nucleotide variants in modulation of blood pressure. This uncovered, amongst others, 87 rare variant-blood pressure associations, establishing the role of rare variants in modulation of this trait in the general population. Their identification has highlighted potential causal pathways and therapeutic targets.

In summary, the thesis provided several insights into the genetic architecture of cardiovascular phenotypes and uncovered novel genes. It is hoped that this knowledge will contribute to clinical implementation of genetic testing for improved care of patients with cardiovascular disorders.

Najim Lahrouchi, Amsterdam UMC, AMC Heart Center, Department of Clinical and Experimental Cardiology, Amsterdam, the Netherlands, (✉) n.lahrouchi@amsterdamumc.nl

Vincent Aengevaeren won the first prize, Najim Lahrouchi the second prize and Martijn Smulders the third prize.

